# Is overweight associated with health-related quality of life (HRQoL) among Tehranian school children?

**DOI:** 10.1186/s40064-016-1930-1

**Published:** 2016-03-10

**Authors:** Sara Jalali-Farahani, Shahram Alamdari, Mehrdad Karimi, Parisa Amiri

**Affiliations:** Research Center for Social Determinants of Endocrine Health and Obesity Research Center, Research Institute for Endocrine Sciences, Shahid Beheshti University of Medical Sciences, P.O.Box: 19395-4763, Tehran, I. R. Iran; Medical Research Development Research Center and Obesity Research Center, Research Institute for Endocrine Sciences, Shahid Beheshti University of Medical Sciences, Tehran, I. R. Iran; Department of Epidemiology and Biostatistics, School of Public Health, Tehran University of Medical Sciences, Tehran, I. R. Iran

**Keywords:** Children, Iran, Quality of life, Obesity, Overweight

## Abstract

This study aimed to determine the association between overweight and health-related quality of life (HRQoL) in Tehranian school children. A total of 631 school children aged 8–14 year old were selected from elementary and secondary schools, and body weight status was determined according to WHO growth references for 5–19 year old children. Children were categorized into the overweight and non-overweight groups. The HRQoL was assessed using Iranian versions of Pediatric Quality of Life Inventory version™ 4.0 (PedsQL). Overweight elementary school boys had significantly higher scores for physical functioning, school functioning and total HRQoL, compared to non-overweight ones (p < 0.05). On the other hand, except for emotional functioning subscale, overweight secondary school boys had significantly lower HRQoL scores, compared to their non-overweight counterparts (p < 0.05). In girls, only social functioning subscale scores were significantly lower in elementary school girls compared to non-overweight ones (p < 0.05). Based on parents’ perspective, overweight elementary school boys had poorer HRQoL compared to their non-overweight counterparts, significant only for emotional functioning (p < 0.05). For secondary school boys, overweight boys had lower HRQoL scores compared to non-overweight ones, significant for all subscales except for emotional functioning. Based on parents’ reports, in both elementary and secondary school girls, there were no significant differences in HRQoL scores between overweight and non-overweight groups. To conclude, in boys while overweight significantly increased HRQoL in children, it significantly decreased HRQoL among adolescents. However, except for social functioning in elementary school girls, there was no significant association between HRQoL scores and overweight. Parents’ reports showed an association between overweight and HRQoL, only in boys.

## Background

The prevalence of overweight and obesity in children and adolescents has risen in both developed and developing countries in recent decades (Wang and Lobstein 2006). Similarly, findings from a national survey of Iranian school children (6–18 years) indicated that about one in five students (20.2 % of girls and 22.9 % of boys) were either overweight or obese (Kelishadi et al. 2013). Several factors in children found to be associated with weight gain, were categorized into three main categories: Genetic, behavioral and environmental factors. In addition to genetic factors which increase the children’s susceptibility to obesity, behavioral factors including unhealthy eating habits and dietary pattern, sedentary lifestyle and lack of physical activity were also found to be associated with overweight and obesity in children. Moreover, the environmental factors (parents, peer, school and community) can per se influence children’s dietary intake and physical activity and consequently their weight status (Karnik and Kanekar 2012). The increase in childhood overweight and obesity is attributable to the shift towards lifestyles characterized by increased intake of energy-dense foods, decreased physical activity levels due to the increasingly sedentary nature of many forms of recreation, changing modes of transportation, and increasing urbanization as well as changes due to socio- economic development and related policies in various sectors of agriculture, education, transportation, urban planning, the environment, food processing, distribution and marketing (World Health Organization).

Overweight and obese children experience more health complications than lean children of the same age (Reilly et al. 2003). Overweight and obesity are reported to be associated with increased risk of development of cardiovascular diseases, high blood pressure, dyslipidemia, type 2 diabetes, and asthma during childhood (Friedemann et al. 2012; Ho 2009; l’Allemand-Jander 2010; Schiel et al. 2006; Gupta et al. 2012; Papoutsakis et al. 2013). Health consequences of overweight and obesity are not just limited to physical health; overweight and obese children experience problems including body dissatisfaction, negative body image, low self-esteem, depression, stigmatization and social marginalization which can influence their psychological and social health issues (Wardle and Cooke 2005; Apolloni et al. 2011). Furthermore, there is evidence indicating obese children and adolescents have impaired HRQoL (Ul-Haq et al. 2013; Ottova et al. 2012; Keating et al. 2011).

There is a general agreement on conceptualization of HRQoL as a multidimensional construct which encompasses at least three main dimensions including the physical, psychological and social well-being of an individual, three dimensions delineated using the World Health Organization (WHO) definition for health (World Health Organization, 1948). In previous studies, sex and age were found to be among factors associated with HRQoL scores in children (Michel et al. 2009). According to existing literature, except for sex and age, other factors including socio-economic status, depressive symptoms, negative self-image, social support, peer victimization and teasing as well as other chronic conditions were found to be associated with the HRQoL of children and adolescents (Stern et al. 2007; Janicke et al. 2007; Zeller and Modi 2006; Di Blasi et al. 2015). In this context, according to children self-reports, obesity was one of the chronic conditions which impaired overall HRQoL even more than certain chronic diseases (Varni et al. 2007). In both community and clinical settings, obese children and adolescents have impaired HRQoL, compared to their normal weight counterparts (Ottova et al. 2012; Keating et al. 2011; Riazi et al. 2010; Wallander et al. 2013; Hughes et al. 2007); the HRQoL impairment in overweight and obese children has been frequently addressed in different domains of HRQoL depending on the sex, age and degree of obesity of the subjects (Ottova et al. 2012; Keating et al. 2011; Riazi et al. 2010; Wallander et al. 2013; Hughes et al. 2007). According to findings of a review by Tsiros et al. physical and social functioning were subscales most affected by childhood overweight and obesity, followed by the emotional functioning subscale (Tsiros et al. 2009).

To our knowledge, few studies have investigated the associations of HRQoL with overweight and obesity among Iranian children; of these two, one including 9–11 year old children (Khodaverdi et al. 2011) and the other 14–17 year old Tehranian adolescents (Jalali-Farahani et al. 2014). Both studies reported poorer HRQoL in overweight or obese children compared to their normal weight counterparts (Khodaverdi et al. 2011; Jalali-Farahani et al. 2014). As mentioned earlier, age is one of the determinants of HRQoL in children (Michel et al. 2009), whereas, early adolescence (the ages 11–14 years) is very important period accompanied by pubertal development, cognitive changes, desire for autonomy, change in relationships with peers and family and educational transition from elementary to high school (Eccles 1999); most existing studies available regarding overweight and HRQoL in Tehranian children are limited to 9–11 and 14–17 year old children (Khodaverdi et al. 2011; Jalali-Farahani et al. 2014). Pre-adolescence is the time that children start to show concern regarding their body due to rapid pubertal changes. Therefore, it is important to show the association between overweight and HRQoL during this age period. Considering lack of evidence regarding this association in Tehranian children during early adolescence, this study aims to investigate the association between overweight and HRQoL in Tehranian school children, aged 8–14 years and to compare this association between elementary and secondary school students.

## Methods

### Participants

Participants of this study were 631 school children, aged 8–14 years, who resided in the north of Tehran; using a simple random sampling method, one district was selected from this area and after preparing lists of all elementary and secondary schools in the selected district, four schools (two elementary and two secondary) were randomly selected. All students in the selected schools were invited to participate in the study.

### Instruments

Body weight and height of children were measured according to standard protocols by trained researchers. Body mass index-for-age (BMI Z score) was determined using Anthroplus software and body weight status of children was determined according to the World Health Organization (WHO) growth references for 5–19 year old children (de Onis et al. 2007). Participants were categorized into two groups, the non-overweight and the overweight; the former included underweight and normal weight children, while the latter included overweight and obese children. The Pediatric Quality of Life Inventory version™ 4.0 (PedsQL) was used to assess HRQoL of students (Varni et al. 2001); this is a multidimensional questionnaire which encompasses two reports: child self-report and parent proxy-report; the first was completed by the child and the second was completed by parents. Both reports consists of four subscales including physical functioning, emotional functioning, social functioning and school functioning. The questionnaire has 23 items; the respondent chooses his/her answer from five response choices ranging from 0 (never a problem) to 4 (almost always a problem). To calculate the subscale and overall scores, the 0–4 scale items was transformed to 0–100 as follows: 0 = 100, 1 = 75, 2 = 50, 3 = 25, 4 = 0; higher scores hence indicate better HRQoL. The reliability and validity of the Iranian version of PedsQL have been reported previously (Amiri et al. 2010, 2012).

### Procedure

Prior to data collection, ethics approval was obtained from the ethics committee of the Research Institute for Endocrine Sciences of Shahid Beheshti University of Medical Sciences in Iran. Approvals were obtained from Ministry of Education and selected schools; following confirmed coordination with school principals. The aims of the study and its procedure was briefly explained for the students; information sheets were then distributed along with parental consent forms and parent proxy-report of PedsQL among the students, providing information about the study aims and its procedure for the parents. The students were asked to pass on the information sheets to their parents, those parents who agreed to participate in the study signed the parental consent form and completed the PedsQL proxy-report, and returned the forms via students to the researchers. After collecting parental forms, those students, whose parents had completed the consent and PedsQL questionnaire were asked to sign consent forms. The students were then invited for anthropometric measurements and were interviewed by the researchers to complete the child-self report of PedsQL questionnaire.

#### Ethical standards

All procedures followed were in accordance with the ethical standards of the responsible committee on human experimentation (The institutional ethics committee of the Research Institute for Endocrine Sciences, Shahid Beheshti University of Medical Sciences, Tehran, Iran) and with the Helsinki Declaration of 1975, as revised in 2000. Informed consents were obtained from both children and their parents for inclusion in the study.

### Statistical analysis

Mean and standard deviation of continues variables and frequency (percentage) of categorical variables were reported as descriptive statistics. Using Multivariate Analysis of Variance (MANOVA) model, the effect of sex, BMI (non-overweight/overweight) and educational level (elementary/secondary) were tested on HRQoL subscale scores. In this model the HRQoL subscale scores considered as dependent variables and sex, BMI and educational level considered as independent factors. The main effects of these factors and their two-way interaction effects were tested on HRQoL subscale scores at mentioned model. Similar univariate model was used for the HRQoL total score, in which, the HRQoL total score was considered dependent variable. Both models described above were applied for self-reports and proxy-reports. Overweight condition (non-overweight/overweight) was compared between sex groups using Chi square test. Using independent t test the means of HRQoL subscales and total scores were compared at sex, BMI and education level groups.

The normality of HRQoL subscales and total scores was checked using Shapiro–Wilk and Kolmogorov–Smirnov tests. Where data were not normally distributed, natural logarithm or SQRT transformations were performed. The SPSS software (version 20.0) was used for analyzing data and significant level was set at 0.05.

## Results

Mean age, BMI-for-age and body weight status of students are presented in Table 1. The total prevalence of severe thinness and thinness in boys and girls were 1.8 and 3.0 % respectively; 39.5 and 35.2 % of boys and girls were overweight and obese respectively.

**Table 1:** Mean of age and body mass index-for-age and body weight status of participants

	Boys (n = 230)	Girls (n = 401)	Total (n = 631)
Age (years)	11.05 ± 2.09	11.00 ± 1.99	11.02 ± 2.02
BMI-for-age	0.67 ± 1.34	0.49 ± 1.26	0.55 ± 1.29
Body weight status			
Severe thinness (%)	0.9	1.0	1.0
Thinness (%)	0.9	2.0	1.6
Normal weight (%)	58.7	61.8	60.6
Overweight (%)	20.9	24.2	23.0
Obese (%)	18.6	11.0	13.8

Using Multivariate Wilk’s-Lambda test for self-reported HRQoL subscale scores, interaction effects were statistically significant for sex*education level (p < 0.001) and education level*overweight (p < 0.05). In the tests of between subject effects, the interaction effect of sex*education level was significant for physical functioning and education level*overweight interaction effect was significant for physical functioning and school functioning (p < 0.001). In the univariate model for self-reported HRQoL total score, sex effect and education level*overweight interaction effect were significant (p < 0.001).

For HRQoL subscale scores of parents’ proxy-reports, using Wilk’s Lambda test, the effects of sex, education level and overweight were significant (p < 0.001) and there was no significant two-way interaction effects for factors. In the tests of between subject effects, the effect of sex was significant on social and school functioning (p < 0.001), the effect of education was significant on physical, emotional and social functioning (p < 0.001) and also the BMI were significantly affected on all HRQoL subscales (p < 0.05). In the univariate model for parents’ proxy-report HRQoL total score, the effect of education level (p < 0.001) and interaction effect of sex*overweight (p < 0.05) were significant. Therefore, more to interpret the effects of sex, BMI and education, subgroup analysis performed to compare HRQoL scores at each category of factors. The mean of HRQoL was compared between overweight groups in each category of sex and education levels separately.

Mean HRQoL subscales and total scores reported by both children and parents are presented in Table 2. According to self-reported HRQoL, both elementary school boys and girls had the lowest HRQoL score in the emotional functioning subscale. Similarly, secondary school boys and girls had the lowest HRQoL scores in emotional functioning subscale. Elementary school girls reported significantly higher HRQoL scores in physical functioning, emotional functioning, social functioning and total HRQoL, compared to boys. Secondary school girls also reported significantly higher HRQoL scores in social functioning, school functioning and total HRQoL, compared to their male counterparts (Table 2).

**Table 2:** Means subscales and total scores of HRQoL reported by children and parents in boys and girls

	Children self-report		p value	Parents’ proxy-report		p value
	Boys	Girls		Boys	Girls	
Elementary school						
Physical functioning	82.84 ± 11.96	87.90 ± 11.91	<0.001*	75.48 ± 17.91	76.18 ± 17.87	0.764
Emotional functioning	80.05 ± 13.60	83.08 ± 15.83	0.009*	70.36 ± 16.91	69.77 ± 16.31	0.703
Social functioning	81.36 ± 15.82	88.91 ± 15.26	<0.001*	75.17 ± 20.57	82.23 ± 16.20	<0.001*
School functioning	81.59 ± 14.38	84.64 ± 12.24	0.095	75.03 ± 16.64	78.34 ± 13.81	0.175
HRQOL total score	81.64 ± 10.12	86.37 ± 9.93	<0.001*	74.27 ± 13.81	76.50 ± 12.59	0.243
Secondary school						
Physical functioning	86.97 ± 11.33	87.76 ± 10.87	0.560	82.71 ± 13.37	86.05 ± 13.16	0.026*
Emotional functioning	76.78 ± 17.98	79.81 ± 16.34	0.127	71.96 ± 19.22	75.11 ± 17.29	0.206
Social functioning	86.59 ± 15.10	94.79 ± 8.39	<0.001*	79.58 ± 18.32	88.37 ± 14.33	<0.001*
School functioning	80.78 ± 15.03	86.14 ± 13.29	<0.001*	75.58 ± 16.34	83.03 ± 14.45	<0.001*
HRQOL total score	83.35 ± 11.46	87.17 ± 9.27	<0.001*	78.14 ± 12.55	83.53 ± 10.78	<0.001*

* Significant

According to parents proxy-reports, in elementary school boys and girls the lowest HRQoL score was reported in emotional functioning subscale (Table 2). Similarly, based on parents’ perspective, both boys and girls in secondary schools had the lowest HRQoL score in emotional functioning subscale. Besides, in elementary schools, girls had higher HRQoL score in social functioning subscale, compared to boys; however, based on parents’ reports, girls in secondary schools had higher HRQoL scores compared to boys in all subscales of HRQoL, except for the emotional functioning subscale.

Table 3 indicates the comparison of self-reported subscale and total scores of HRQoL between non-overweight and overweight school children. In elementary school children, overweight boys had higher subscale and total HRQoL scores compared to non-overweight boys, with significant differences in physical functioning, school functioning and total HRQoL scores. However, in elementary school girls, no significant differences were seen in subscale and total scores of HRQoL between non-overweight and overweight groups except for social functioning which was significantly higher in non-overweight girls, compared to their overweight counterparts. In secondary school children, overweight boys had lower subscale and total HRQoL scores, compared to non-overweight counterparts, significant for all scores except for emotional functioning. As for girls, overweight girls had lower scores compared to their non-overweight counterparts in all subscales and total HRQoL scores, differences not statistically significant.

**Table 3:** Comparison of child self-reported means subscales and total scores of HRQoL by overweight

	Non-overweight	Overweight	p value
Elementary school (n = 321)			
Boys (n = 110)	n = 65	n = 45	
Physical functioning	80.58 ± 12.44	86.11 ± 10.50	0.026*
Emotional functioning	79.08 ± 15.15	81.44 ± 11.01	0.971
Social functioning	79.31 ± 17.02	84.33 ± 13.55	0.198
School functioning	79.15 ± 15.14	85.11 ± 12.54	0.031*
HRQOL total score	79.67 ± 10.89	84.49 ± 8.19	0.038*
Girls (n = 211)	n = 138	n = 73	
Physical functioning	87.89 ± 12.11	87.93 ± 11.60	0.901
Emotional functioning	82.79 ± 16.09	83.63 ± 15.44	0.948
Social functioning	90.54 ± 13.53	85.82 ± 17.78	0.026*
School functioning	84.64 ± 11.76	84.66 ± 13.18	0.631
HRQOL total score	86.66 ± 9.44	85.82 ± 10.84	0.782
Secondary school (n = 310)			
Boys (n = 120)	n = 74	n = 46	
Physical functioning	89.71 ± 9.91	82.56 ± 12.17	<0.001*
Emotional functioning	80.34 ± 13.61	71.06 ± 22.37	0.072
Social functioning	89.38 ± 11.55	82.17 ± 18.76	0.025*
School functioning	83.31 ± 12.96	76.70 ± 17.24	0.024*
HRQOL total score	86.21 ± 9.32	78.74 ± 13.08	<0.001*
Girls (n = 190)	n = 122	n = 68	
Physical functioning	88.39 ± 9.46	86.63 ± 13.02	0.726
Emotional functioning	80.73 ± 15.29	78.16 ± 18.06	0.520
Social functioning	95.20 ± 8.37	94.04 ± 8.43	0.157
School functioning	87.27 ± 13.08	84.12 ± 13.52	0.060
HRQOL total score	87.91 ± 8.52	85.86 ± 10.42	0.306

* Significant

Table 4 compares parent proxy-reported subscale and total scores of HRQoL between non-overweight and overweight school children. Based on parents’ perspectives, in elementary school children, overweight boys had lower subscales and total HRQoL scores compared to non-overweight boys, with a significant difference for emotional functioning. However, in girls no significant differences were observed between non-overweight and overweight groups. In secondary school children, overweight boys had lower subscales and total HRQoL scores compared to non-overweight boys, differences significant for all subscales, except for emotional functioning. Among girls, except for the emotional functioning subscale, overweight girls had lower scores compared to their non-overweight counterparts, differences not statistically significant.

**Table 4:** Comparison of parent proxy-reported means subscales and total scores of HRQoL by overweight

	Non-overweight	Overweight	p value
Elementary school (n = 321)			
Boys (n = 110)	n = 65	n = 45	
Physical functioning	76.59 ± 18.05	73.91 ± 17.79	0.393
Emotional functioning	73.54 ± 16.20	65.78 ± 17.05	0.019*
Social functioning	76.21 ± 2.43	73.67 ± 19.41	0.341
School functioning	76.85 ± 16.60	72.42 ± 16.54	0.175
HRQOL total score	75.96 ± 13.98	71.86 ± 13.37	0.127
Girls (n = 211)	n = 138	n = 73	
Physical functioning	76.77 ± 18.51	75.07 ± 16.64	0.327
Emotional functioning	70.04 ± 16.78	69.26 ± 15.50	0.597
Social functioning	82.28 ± 16.42	82.12 ± 15.88	0.929
School functioning	78.42 ± 13.32	78.19 ± 14.81	0.944
HRQOL total score	76.73 ± 12.91	76.08 ± 12.02	0.638
Secondary school (n = 310)			
Boys (n = 120)	n = 74	n = 46	
Physical functioning	85.74 ± 12.76	77.83 ± 13.00	<0.001*
Emotional functioning	73.72 ± 19.05	69.13 ± 19.36	0.202
Social functioning	82.77 ± 17.30	74.46 ± 18.92	0.016*
School functioning	78.04 ± 15.89	71.63 ± 16.43	0.035*
HRQOL total score	80.84 ± 12.33	73.81 ± 11.76	<0.001*
Girls (n = 190)	n = 122	n = 68	
Physical functioning	86.26 ± 11.78	85.67 ± 15.42	0.908
Emotional functioning	74.85 ± 17.15	75.59 ± 17.65	0.725
Social functioning	88.98 ± 13.45	87.28 ± 15.82	0.579
School functioning	83.98 ± 12.97	81.34 ± 16.75	0.380
HRQOL total score	83.88 ± 9.66	82.89 ± 12.58	0.861

* Significant

## Discussion

This study aimed to examine the association between overweight and HRQoL among school-aged Tehranian youth; considering the influence of sex and age on HRQoL of children, the aforementioned association was assessed in different sex and age groups. The prevalence of underweight (2.6 %) was less than overweight (23.0 %) and obesity (13.8 %), indicating that overweight was a more serious health concern than underweight in our study population, a finding confirming previous reports regarding high prevalence rates and increasing trend of overweight and obesity in Tehranian children (Hosseini-Esfahani  et al. 2011).

### HRQoL in sex and age groups

Our results showed that, as a whole, girls had better HRQoL compared to boys. At younger ages (8–11 years), higher HRQoL scores were observed, specifically in the physical, emotional and social functioning subscales; in older age (11–14 years), higher HRQoL scores were observed in social and school functioning subscales of HRQoL. However, a previous study conducted among Tehranian high school students (14–17 years), indicated that girls had lower HRQoL scores compared to boys (Jalali-Farahani et al. 2014); comparing the two findings indicates that with increasing age, the HRQoL total score decreases in girls, while it has an increasing trend in boys, a decrement in girls, which may be due to pubertal changes. In a qualitative study, many Iranian adolescent girls reported that puberty was an unpleasant experience for them (Golchin et al. 2012). In addition, in a nationwide survey of female Iranian adolescents, Rabbani et al. also reported that increment of age, onset of menstrual cycle, residing in urban areas and ethnic groups were factors associated with mental health problems (Rabbani et al. 2012). Although, adolescent Iranian boys also reported experiencing some degree of negative feelings such as shame, embarrassment and anxiety during puberty (Ahmadi et al. 2009), it seems puberty is accompanied by more sudden and severe changes in girls, than in boys (Benjet and Hernández-Guzmán 2002) which may contribute to deterioration in HRQoL observed with increasing age in girls, compared to boys.

### Overweight and self-reported HRQoL

Findings of the current study indicate that the association between self-reported HRQoL and overweight followed different patterns in younger and older boys. While overweight elementary school boys (8–11 years) reported better HRQoL compared to their non-overweight counterparts; adolescent boys (11–14 years) reported poorer HRQoL compared to normal weight ones. Findings of current study regarding the association between overweight and HRQoL in adolescent boys is consistent with previous findings among 14–17 year old Tehranian boys (Jalali-Farahani et al. 2014). On the other hand, based on findings of the current study, overweight 8–11 year old elementary school girls had significantly lower HRQoL scores only in social functioning, compared to non-overweight ones, whereas no significant differences were observed in HRQoL scores between overweight and non-overweight adolescent girls (11–14 years). Previous findings in 14–17 year old Tehranian girls, indicate lower HRQoL scores in overweight girls, compared to their normal weight counterparts (Jalali-Farahani et al. 2014). Considering both the findings of this study and a previous one conducted among high school students (Jalali-Farahani et al. 2014), it seems that the association between overweight and HRQoL follows different patterns based on the adolescent’s sex and age. Higher HRQoL scores in overweight elementary school boys, compared to their normal weight counterparts in current study are consistent with those of a population based study, conducted in Fiji (Petersen et al. 2014); Petersen et al. reported that obese 12–14 year old Fijian children had higher HRQoL scores, compared to their normal weight counterparts, whereas in 15–18 year old children, normal weight children had better HRQoL scores compared to the obese group (Petersen et al. 2014). However, another study conducted among primary school Tehranian children (Khodaverdi et al. 2011), reported poorer HRQoL in obese children, compared to normal weight ones, findings inconsistent with ours regarding elementary school boys, this inconsistency may be due to not conducting sex specific analysis in the mentioned study (Khodaverdi et al. 2011). However, there is consensus in reporting lower HRQoL scores in overweight or obese children, regardless of their age and sex, compared to their normal weight counterparts in most previous studies (Ottova et al. 2012; Keating et al. 2011; Riazi et al. 2010; Wallander et al. 2013; Hughes et al. 2007). In Tehranian children the association between overweight and HRQoL follows different patterns depending on sex and age, patterns which may be due to cultural differences; for further clarification, according to the findings of a qualitative research conducted among Tehranian adolescents, some overweight/obese adolescents, viz. boys, had positive self-images accompanied by believing in higher resistance to illness and physical blows, compared to their normal weight peers as well as believing in their equal ability to compete in sports and physical activities with their normal weight peers, neither did number of participants perceive overweight/obesity as a threat to their health (Amiri et al. 2011). This positive self-image and its related beliefs as well as perceived lack of threat due to overweight/obesity may contribute to higher HRQoL scores reported by some overweight children, a hypothesis which needs to be further investigated.

Other factors that can also mediate the association between weight status and HRQoL; there is evidence indicating that compared to actual weight status, the child’s own perception of being overweight may be a better predictor of psychosocial problems and unhealthy weight control behaviors (Armstrong et al. 2014). According to the Armstrong et al. study (2014), there was no significant correlation between BMI and depressive symptoms, whereas perception of overweight and depressive symptoms were significantly correlated. In addition, a recent study showed that weight perception moderates the association between overweight/obesity and HRQoL in adolescents, as overweight adolescents who perceived themselves to be overweight reported lower HRQoL, compared to those overweight ones who perceived themselves as normal weight (Hayward et al. 2014). Misperception regarding body weight status is a common issue among children and adolescents, with higher prevalence in former group compared to the latter (Heshmat et al. 2015; Sarafrazi et al. 2014). In a nationwide population-based survey of Iranian children (10–18 years), weight perception was associated with both life satisfaction and self-rated health (Heshmat et al. 2015). Some studies indicate that compared to normal weight adolescent boys, more normal weight adolescent girls tend to misperceive themselves as overweight (Sarafrazi et al. 2014; Kim and So 2014), which may be due to different perceptions of ideal body size and shape in these groups, girls preferring smaller and slimmer bodies, compared to larger and muscular bodies in boys (Cohane and Pope 2001). Hence, in comparison to boys, more normal weight girls may have misperception regarding their body weight statuses and incorrectly perceive themselves as overweight or obese, which may result in dissatisfaction regarding their body weight and shape, and consequently more unhealthy control behaviors, experiencing of negative feelings and adverse outcomes (Armstrong et al. 2014; Johnson and Wardle 2005), all of which may contribute to decrement in their HRQoL scores, irrespective of their actual body weight status and consequently confound the association between overweight and HRQoL in our study. Over-valuation of weight and shape can be another factor influencing HRQoL specifically among girls. Data indicating that those girls who tend to do this are more prone to develop binge eating, compared to their less weight concerned peers and weekly binge eating was found to be associated with more depressive symptoms and lower subjective social status (Sonneville et al. 2015). Furthermore, based on data available, self-image is a factor that influences interpersonal relations and is associated with social anxiety disorder (Di Blasi et al. 2015). The aforementioned findings imply that overvaluation of weight and shape and one’s self-image are also contributing factors to one’s psychosocial functioning irrespective of weight status, indicating that there are other factors which may independently influence HRQoL in children or mediate the association between overweight and HRQoL. It is hence recommended to investigate the potential role of body image and its related components on children’s HRQoL in future studies aiming at exploring the association between body weight status and HRQoL in children.

### Overweight and parent-reported HRQoL

Using parents’ perspectives, HRQoL scores were lower in overweight boys, irrespective of their age group; besides, more subscales were significantly impaired in older boys (11–14 years) compared to younger ones (8–11 years). The comparison between boys’ perceptions and parents’ perceptions regarding the association between overweight and HRQoL scores indicated that elementary school boys’ perspectives differ from their parents, whereas those of secondary school boys’ were very similar to their parents. Furthermore, based on parents’ perceptions, in both elementary and secondary school girls no significant differences in HRQoL scores were found between overweight and non-overweight groups which is very similar to their children. Previous studies report good agreement between children self-reports and parents proxy-reports of HRQoL in Tehranian children (Amiri et al. 2010, 2012) which are consistent with our findings regarding similar perspectives between children and parents in elementary school girls and secondary school boys and girls.

### Strengths and limitations of the study

To the best of our knowledge, this is the first time that the association between overweight and HRQoL has been reported in Tehranian children during early adolescence. However, the limitations for this study should be noted. Due to the cross-sectional nature of this study, we cannot make causal inferences about associations between overweight and HRQoL. Secondly, in the current study BMI was used to determine body weight status of the students, although BMI cannot distinguish between fat and muscle mass and it does not precisely indicate the distribution of body fat; hence, BMI may not be an accurate measure of body fat and indicator of healthy body weight. Moreover, the participants of this study were limited to a representative sample of children residing in north of Tehran, but not the whole city. Hence, to confirm our findings and get more conclusive results, conducting studies that include students from other areas of Tehran is recommended. Finally, there are other important factors that may influence HRQoL in children and adolescents which were not included in the current study; further research is needed to investigate factors associated with poorer HRQoL in this age groups.

## Conclusion

In conclusion, in boys while overweight could significantly increase HRQoL in children, it significantly decreased HRQoL among adolescents. However, none of the total HRQoL and subscale scores were significantly associated with overweight except for social functioning in elementary school girls. Parents reported impairment in HRQoL only in boys. HRQoL can serve as a beneficial index for monitoring different dimensions of health in overweight/obese children. Information regarding the impaired domains of HRQoL among overweight and obese children would provide beneficial information for designing interventions aiming at improving children’s lifestyles and their HRQoL. Additionally, obtaining perspectives of parents and incorporating them in these interventions would increase the effectiveness of health promotion programs in children.
